# Neolignans isolated from twigs of *Nectandra leucantha* Ness & Mart (Lauraceae) displayed in vitro antileishmanial activity

**DOI:** 10.1186/s40409-018-0164-9

**Published:** 2018-09-27

**Authors:** Simone S Grecco, Thais A Costa-Silva, Fernanda S Sousa, Stefano B Cargnelutti, Eric Umehara, Poliana S Mendonça, Andre G Tempone, Joao Henrique G Lago

**Affiliations:** 1 Center of Natural Sciences and Humanities, Federal University of ABC (UFBC), Avenida dos Estados 5001, Santo Andre, SP 09210-580 Brazil; 2 Biotechnology and Innovation in Health Postgraduate Program, Anhanguera University of São Paulo, São Paulo, SP Brazil; 3 Institute Environmental, Chemical and Pharmaceutical Sciences, Federal University of São Paulo (UNIFESP), Diadema, SP Brazil; 4 Center for Parasitology and Mycology, Instituto Adolfo Lutz, São Paulo, SP Brazil

**Keywords:** *Nectandra leucantha*, *Leishmania* (L.) *infantum*, Antileishmanial activity, Neolignans

## Abstract

**Background:**

The therapeutic arsenal for the treatment of Leishmaniasis is limited and includes toxic compounds (antimonials, amphotericin B, pentamidine and miltefosine). Given these aspects, the search for new compounds based on floristic biodiversity is crucial. In the present work, we report the isolation, characterization and antileishmanial activity of six related neolignans (**1–6**) of bioactive extract from *Nectandra leucantha* (Lauraceae) twigs.

**Methods:**

Dried and powdered twigs of *N. leucantha* were exhaustively extracted using *n*-hexane. The crude extract was dereplicated by HPLC/HRESIMS and subjected to column chromatography to yield pure compounds **1–6**. Their chemical structures were identified via NMR and comparison of obtained data with those previously published in the literature. Biological assays of compounds **1–6** and their respective monomers (eugenol and methyleugenol) were performed using promastigote and amastigote forms of *Leishmania* (L.) *infantum*.

**Results:**

Dereplication procedures followed by chemical characterization of isolated compounds by NMR enabled the identification of related neolignans **1–6**. Neolignans **2**, **4** and **6** showed potential against amastigote forms of *L.* (L.) *infantum* (EC_50_ values of 57.9, 67.7 and 13.7 μM, respectively), while compounds **1** and **3** were inactive. As neolignans **2–4** are chemically related, it may be suggested that the presence of the methoxyl group at C4 constitutes an important structural aspect to increase antileishmanial potential against amastigote forms. Compound **6**, which consists of a methylated derivative of compound **5** (inactive) showed antileishmanial activity similar to that of the standard drug miltefosine (EC_50_ = 16.9 μM) but with reduced toxicity (SI = 14.6 and 7.2, respectively). Finally, two related monomers, eugenol and methyleugenol, were also tested and did not display activity, suggesting that the formation of dimeric compounds by oxidative coupling is crucial for antiparasitic activity of dimeric compounds **2**, **4** and **6**.

**Conclusion:**

This study highlights compound **6** against *L.* (L.) *infantum* amastigotes as a scaffold for future design of new compounds for drug treatment of visceral leishmaniasis.

## Background

Leishmaniasis is a neglected tropical disease (NTD) that affects more than one billion people in tropical and subtropical countries, including parts of Latin America, Africa and Asia [1, 2]. This disease is caused by protozoa of the genus *Leishmania* and transmitted by the bite of infected sandflies. The World Health Organization estimated 1.3 million new cases annually, divided into 300,000 cases of visceral leishmaniasis and 1 million cases of cutaneous leishmaniasis. [2]. The therapeutic arsenal for the treatment of leishmaniasis is limited and still unsatisfactory. The treatment is not only challenging and long, but also offers a reduced option of drugs, most of which are toxic such as antimonials, amphotericin B, pentamidine and miltefosine [3, 4]. Considering this problematic context and lack of interest from the pharmaceutical sector, the search for novel drugs is crucial [5]. Based on this aspect, natural products may be considered an interesting source of new molecules for the development of scaffolds for protozoal diseases, including leishmaniasis. Previous studies by our group performed on the *n*-hexane extract from twigs of *Nectandra leucantha* (Lauraceae) yielded three neolignans with significant antileishmanial and immunomodulatory activity against *Leishmania* (L.) *donovani* [6]. Additionally, these related compounds displayed activity against *Trypanosoma cruzi* [7]. As part of our continuous studies of *N. leucantha*, in the present work, the crude *n*-hexane extract from twigs of this plant displayed activity against promastigote and amastigote forms of *L.* (L.) *infantum* (death rate of 100% at 300 μg/mL). Aiming to characterize its bioactive compounds, the crude extract was subjected to dereplication procedures using HPLC/HRESIMS to enable the identification of six related neolignans (**1–6**). Since there was no information concerning the potential of these neolignans against amastigote forms of *L.* (L.) *infantum*, the crude bioactive extract was fractionated using different chromatographic methods to obtain pure **1–6**. Antileishmanial activity and cytotoxicity of each isolated compound was evaluated in vitro and the important chemical features related to the antileishmanial activity of these related natural products were determined.

## Methods

### General

^1^H and ^13^C NMR spectra were recorded at 300 and 75 MHz, respectively, in a Bruker Ultrashield 300 Avance III spectrometer with a QNP 300 (5 mm) probe, using CDCl_3_ (Aldrich) as the solvent and TMS as the internal standard. HRESIMS spectra were recorded on a Bruker MicroTOF-QII using electrospray ionization in the positive mode. Silica gel (Merck, 230–400 mesh) and Sephadex LH-20 (Sigma-Aldrich) were employed for the column chromatography separation, while silica gel 60 PF_254_ (Merck) was used for analytical thin-layer chromatography (TLC). Eugenol and methyleugenol, purchased from Sigma-Aldrich, were purified by distillation. Miltefosine was also purchased from Sigma-Aldrich. HPLC analysis was performed in a Dionex Ultimate 3000 chromatograph with a UVD-DAD 170 V as a detector, using a Luna Phenomenex C18 column (10 × 250 mm, particle and pore size of 5 μm and 175 Å, respectively) in the semi-preparative mode or Dionex C18 (4.6 × 250 mm, particle and pore size of 5 μm and 120 Å, respectively) in the analytical mode.

### Plant material and preparation of crude extract

*N. leucantha* twigs were collected from a single specimen at the Atlantic Forest area of São Paulo state, Brazil, in March 2014. A voucher specimen (EM357) has been deposited in the Herbarium of Institute of Biosciences, University of São Paulo, SP, Brazil. Dried (40 °C at 72 h) and powdered twigs (220 g) of *N. leucantha* were exhaustively extracted using *n*-hexane at room temperature. After concentration under reduced pressure, 7.9 g of crude *n*-hexane extract was obtained.

### HPLC/HRESIMS analysis

Part of crude *n*-hexane extract from twigs of *N. leucantha* (5 mg) was dissolved in MeOH and filtered on a C18 Sep-Pak. HPLC/HRESIMS were recorded on an Shimadzu GC-17A (Kyoto, Japan) HPLC system equipped with a DAD detector. Separation of 10 mg/μL of sample was performed using a Dionex column (4.6 × 250 mm, particle and pore size of 5 μm and 120 Å, respectively) eluted with a gradient from MeOH:H_2_O 1:1 (0 min) to MeOH 100% (35 min), flow rate 1.0 mL/min and detection at 237 nm. MS detection (positive mode) was obtained on a Bruker micrOTOF-QII (Billerica, MA, USA) coupled to an Apollo ion source set as follows: dry temperature at 180 °C and voltage at 4.5 kV. The mass/charge ratios were detected in scan (*m/z* 100–1200 Da) and product ion scan (*m/z* 50–1200 Da) modes.

### Extraction and isolation

Part of *n-*hexane extract from twigs of *N. leucantha* (7 g) was chromatographed over a silica gel column eluted with mixtures of *n*-hexane:EtOAc (1:0, 9:1, 8:2, 7:3, 6:4, 1:1, 3:7, 1:9, and 0:1) to yield ten fractions (A – J). Fraction C (1528 mg) was composed of compound **1** (99% of purity). Fraction E (1928 mg) was ressuspended in MeOH and chromatographed in a Sephadex LH-20 column eluted with MeOH to obtain five fractions (E1 – E5). Fractions E3 (889 mg) and E4 (268 mg) were composed of compound **2** (100% of purity). Fraction G (1450 mg) was chromatographed over a silica gel column eluted with mixtures of *n*-hexane:EtOAc (9:1, 8:2, 7:3, 6:4, 1:1, 3:7, 1:9, and 0:1) to yield eight fractions (G1 – G8). Fraction G3 (386 mg) was chromatographed over a silica gel column eluted with mixtures of *n*-hexane:EtOAc (8:2, 7:3, 1:1, 3:7, 1:9, and 0:1) to obtain five fractions (G3/1 – G3/5). Fraction G3/4 (150 mg) was composed of compound **3** (100% of purity). Fraction G4 (865 mg) was composed of compound **4** (99% of purity). Fraction H (108 mg) was purified by semi-preparative RP-18 HPLC, eluted with MeOH:H_2_O 8:2 (flow rate = 2 mL/min, λ = 237 nm) to yield, respectively, compounds **5** (21 mg - 99% of purity) and **6** (14 mg - 100% of purity).

### Bioassays

BALB/c mice and Golden hamsters were supplied by the animal breeding facility at the Adolfo Lutz Institute, São Paulo, Brazil, and maintained in sterilized cages in a controlled environment, receiving water and food ad libitum.

### Parasites

Isolated promastigotes of *Leishmania* (L.) *infantum* (MHOM/BR/1972/LD) were maintained in M-199 medium supplemented with 10% calf serum and 0.25% hemin at 24 °C. The *L.* (L.) *infantum* (MHOM/BR/1972/LD) was maintained in hamsters (*Mesocricetus auratus*). Amastigotes were harvested from spleens of infected hamsters. The animals were infected with 10^8^ amastigotes (300 μL) by the i.p. route. At 60 days post-infection, the animals were euthanized, and the spleens analyzed by light microscopy using Giemsa-stained smears. The parasite load was expressed in Leishman-Donovan units (i.e., the number of amastigotes per 1000 nucleated cells x organ weight [in g] × 2 × 10^5^). Briefly, the spleen was macerated in a tissue grinder tube containing 5 mL of phosphate-buffered saline (PBS), whereas the amastigotes were separated by differential centrifugation to obtain a suspension of parasites. The first centrifugation of the spleen suspension (0.8 rpm) was employed to separate the red and white cells (pellet) from the amastigote forms (supernatant). Next, a second centrifugation of the supernatant from the previous step (2.8 rpm) was performed to concentrate the amastigote forms [8].

### Mammalian cells

Peritoneal macrophages were collected from the peritoneal cavity of female BALB/c mice by washing with RPMI-1640 without phenol red, supplemented with 10% fetal bovine serum. Murine conjunctive cells (NCTC clone L-929, ATCC) were maintained in RPMI-1640 supplemented with 10% FBS at 37 °C in a humidified atmosphere containing 5% CO_2_ [9]_._

### Determination of the activity against *Leishmania* (L.) *infantum.* - promastigotes

To determine the antileishmanial activity and the 50% effective concentration (EC_50_ value) against promastigotes, crude *n*-hexane extract and pure compounds **1–6** were dissolved in DMSO (30 mg/mL) and diluted with M-199 medium in 96-well microplates. Promastigotes were counted in a Neubauer hemocytometer and seeded at 1 × 10^6^/well to obtain a final volume of 150 μL. Controls with DMSO and without drugs were also performed. Miltefosine was dissolved in H_2_O at 30 mg/mL and used as a standard drug. To determine the antileishmanial potential, crude extract was tested at 300 μg/mL while compounds **1–6** and positive control miltefosine were tested at top concentration 200 μg/mL and were 2-fold serially diluted into seven concentrations (100, 50, 25, 12.5, 6.75, 3.37, and 1.69 μg/mL). Each point was tested in duplicate. The plate was incubated for 48 h at 24 °C and the viability of promastigotes was verified by morphology in light microscopy and by the MTT assay [10]. Briefly, MTT (5 μg/mL) was dissolved in PBS, sterilized through 0.22 μm membranes and added (20 μL/well) for 4 h at 24 °C. Promastigotes were incubated without compounds and used as viability control. Formazan extraction was performed using 10% SDS for 18 h (80 μL/well) at 24 °C and the optical density (OD) was determined in a Multiskan MS (UNISCIENCE) at 550 nm. One hundred percent (100%) viability was expressed based on the OD of control promastigotes, after normalization.

### Determination of the activity against *L.* (L.) *infantum*. - intracellular amastigotes

To determine the 50% effective concentration (EC_50_ value), mice peritoneal macrophages were collected from the peritoneal cavity of BALB/c mice, then seeded at 1 × 10^5^/well for 24 h in a 16-well slide (Nunc – Thermo) at 37 °C. Amastigotes were isolated from spleens of previously infected hamsters, and separated by differential centrifugation [8]. The tissue sample was macerated to break the cell membranes to release all cell contents including the amastigotes. Then the lysate was subjected to two repeated centrifugations (0.8 rpm followed 2.8 rpm) where density separation causes a sediment. The supernatant and the pellet were recovered via the first and second centrifugation, respectively. Then the purified amastigotes were added to the macrophages at a ratio of 1:10 (macrophage/amastigotes) for 24 h at 37 °C. Non-internalized parasites were removed by washing once with medium, whereas tested compounds were incubated with infected macrophages for 96 h. Miltefosine was also used as a standard control drug. Subsequently, the cells were fixed with MeOH, stained with Giemsa and observed through a light microscope. The parasite burden was determined by the ratio of infected macrophages versus the mean number of amastigotes per macrophage out of 500 macrophages, i.e., the parasitic index (PI) [11]. Similarly to the assays conducted to promastigote forms, the potential of crude *n*-hexane extract was evaluated at 300 μg/mL while the activities of compounds **1–6** and positive control miltefosine against amastigote forms of *L. (L.) infantum* were determined using a top concentration at 200 μg/mL and were 2-fold serially diluted into seven concentrations (100, 50, 25, 12.5, 6.75, 3.37, and 1.69 μg/mL). Each point was tested in duplicate.

### Determination of the cytotoxicity against mammalian cells

To determine the 50% cytotoxic concentration (CC_50_ value), NCTC cells-clone 929 (6 × 10^4^ cells/well) were seeded at 4 × 10^4^ cells per well in 96-well microplates and incubated with compounds **1–6** for 48 h at 37 °C in a 5% CO_2_ incubator using a top concentration at 300 μg/mL and were 2-fold serially diluted over seven concentrations (150, 75, 37.5, 18.75, 9.37, 4.69 and 2.34 μg/mL). Each point was tested in duplicate. The selectivity index (SI) was determined using the following ratio: CC_50_ against macrophages/EC_50_ against amastigotes. Cell viability was determined by the MTT assay as described previously [10].

### Statistical analysis

The obtained data represent the mean and standard deviation of duplicate samples from two independent assays. EC_50_ values were calculated using sigmoid dose–response curves in the software GraphPad Prism 5.0; the 95% confidence intervals are included in parentheses. The Mann–Whitney test was used for the significance test (*P* value).

## Results and discussion

**Fig. 1:**
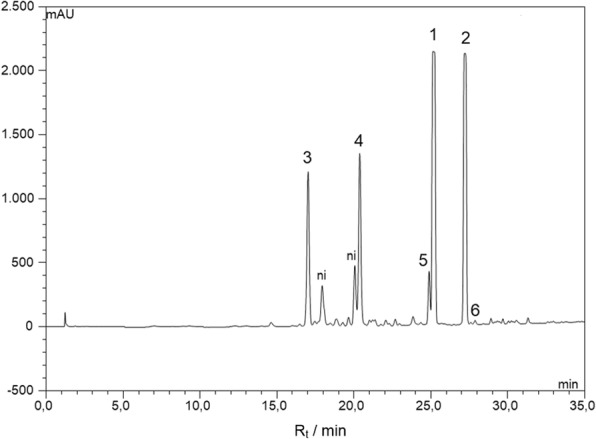
HPLC/HRESIMS analysis of crude n-hexane extract from twigs of *N. leucantha.* Compound **1** *m/z* 349.1423 [M + Na]^+^, compound **2** *m/z* 363.1585 [M + Na]^+^, compound **3** *m/z* 365.1237 [M + Na]^+^, compound **4** *m/z* 379.1506 [M + Na]^+^, compound **5** *m/z* 327.1589 [M + H]^+^, and compound **6** *m/z* 355.1911 [M + H]^+^. ni = non-identified compound

**Fig. 2:**
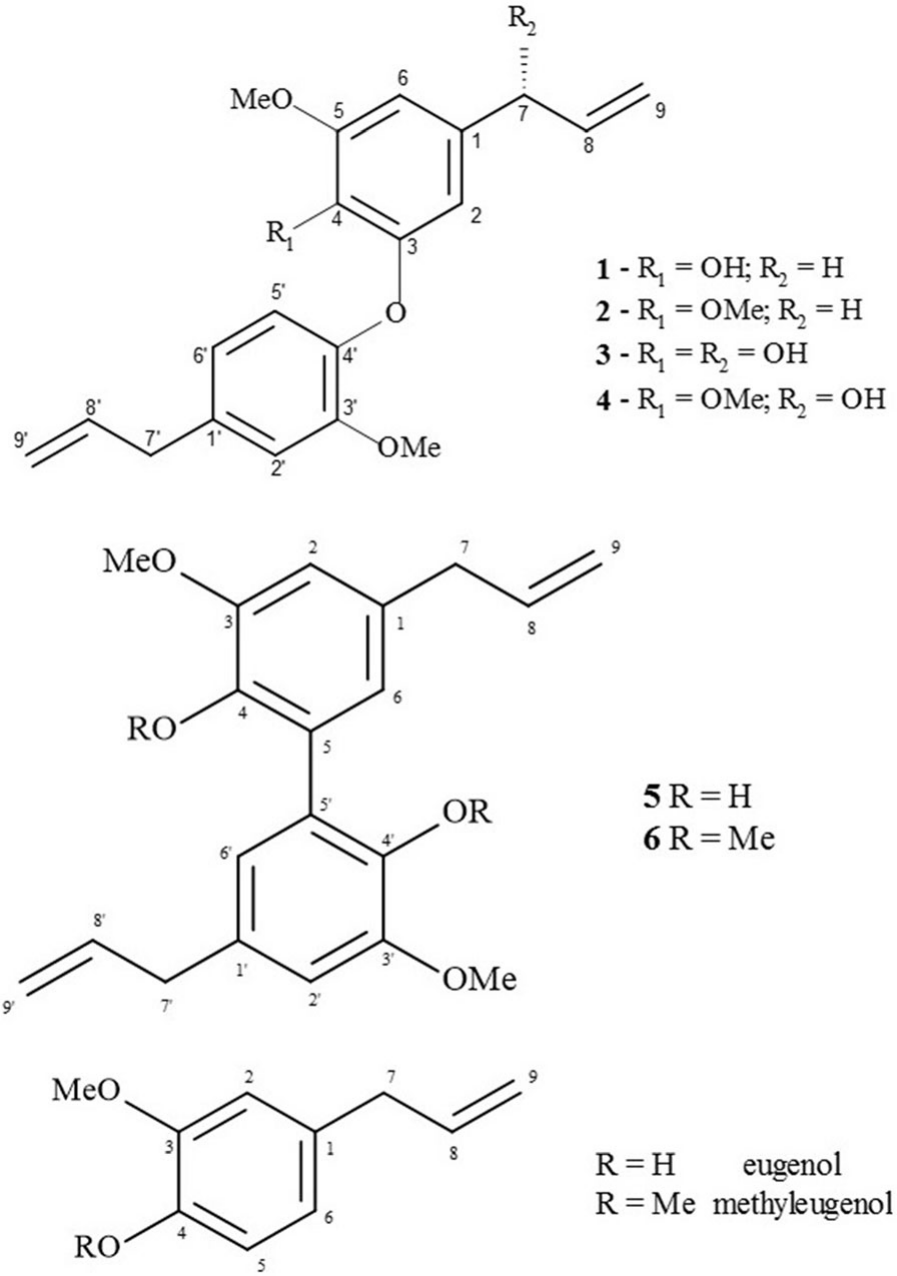
Structures of neolignans **1–6** isolated from *N. leucantha* and monomers eugenol and methyleugenol

The antileishmanial potential of *n*-hexane extract from twigs of *N. leucantha* was evaluated against promastigote and amastigote forms of *L.* (L.) *infantum*; 100% death of parasites was detected at 300 μg/mL. This extract was subjected to the dereplication procedure using HPLC/HRESIMS (Fig. 1), which indicated the occurrence of related neolignans from the *quasi*-molecular ion peaks at *m/z* 349.1423 [M + Na]^+^, 363.1585 [M + Na]^+^, 365.1237 [M + Na]^+^, 379.1506 [M + Na]^+^, 327.1589 [M + H]^+^, and 355.1911 [M + H]^+^, corresponding to molecular formulas C_20_H_22_O_4_ (**1**), C_21_H_24_O_4_ (**2**), C_20_H_22_O_5_ (**3**), C_21_H_24_O_5_ (**4**), C_20_H_22_O_4_ (**5**) and C_22_H_26_O_4_ (**6**). Aiming to obtain bioactive compounds, the crude extract was subjected to several chromatographic steps to yield pure compounds **1–6** which were analyzed by NMR. The ^1^H NMR spectra of compounds **1–4** displayed a profile of neolignan derivatives attributable to signals of hydrogens from aromatic rings at δ 6.90–6.81 (d, *J* = 8.1 Hz, H-5′), 6.79 (d, *J* = 2.0 Hz, H-2′), 6.71–6.69 (dd, *J* = 8.1 and 2.0 Hz, H-6′), 6.70–6.48 (*J* = 1.8 Hz, H-2) and 6.56–6.27 (d, *J* = 1.8 Hz, H-6), to saturated carbon aliphatic hydrogens at δ 3.24–5.04 (d, *J* = 6.6 Hz, H-7) and 3.36–3.39 (d, *J* = 6.7 Hz, H-7′) as well as to hydrogens linked to sp^2^ carbons at δ 5.06–5.98 (m, H-8/H-8′ and H-9/H-9′). Additionally, the spectra of compounds **1** and **3** showed two intense singlets at δ 3.89 (3H) and 3.86 (3H) attributable to methoxyl groups at C-5 and C-3′. In the case of compounds **2** and **4**, two singlets were observed at δ 3.87 (6H) and 3.83 (3H), assigned to methoxyl groups at C-4, C-5 and C-3′. ^13^C NMR spectra of compounds **1–4** displayed ten signals at range of δ 105–153 and two signals at approximately δ 144, which revealed the presence of two aromatic rings linked by an oxygen bond. Additional sp^2^ carbon atoms at δ 116–140 (C-8/C-8′ and C-9/C-9′), associated with the presence of methylene carbon atoms at approximately δ 40 (C-7/C-7′) in **1** and **2**, indicated the presence of two allyl side chains. Otherwise, in the cases of **3** and **4**, signals were observed at approximately δ 75.0 (CH), which were attributed to the carbinolic carbon C-7. The ^1^H NMR spectra of compounds **5** and **6** displayed a similar profile of eugenol attributable to the signals attributed to hydrogens of aromatic rings at δ 6.69–6.73 (d, *J* = 2.0 Hz, H-2/H-2′) and 6.74–6.76 (d, *J* = 2.0 Hz, H-6/H-6′), to saturated carbon aliphatic hydrogens at δ 3.36 (d, *J* = 6.7 Hz, H-7/H-7′), and to hydrogens linked to sp^2^ carbons at δ 5.09–5.98 (m, H-8/H-8′ and H-9/H-9′). Additionally, intense singlets at δ 3.92 (compound **5**) and δ 3.63/3.88 (compound **6**) were assigned to methoxyl groups. The ^13^C NMR spectra of compounds **5** and **6** showed nine signals related to the C_6_-C_3_ structure as well as additional peaks attributable to methoxyl groups at δ_C_ 56.1 to compound **5** and at δ_C_ 56.0/60.8 to compound **6**. The pattern related to a tetrasubstituted aromatic ring confirmed the C-C linkage between two aromatic rings. The spectroscopic data of **1**–**6** were in accordance with those previously reported in the literature [6, 7, 12–18], allowing the identification of dehydrodieugenol B (**1**), 1-(8-propenyl)-3-[3′-methoxy-1′-(8-propenyl)phenoxy]-4,5-dimethoxybenzene (**2**), 1-[(7R)-hydroxy-8-propenyl]-3-[3′-methoxy-1′-(8′-propenyl)-phenoxy]-4-hydroxy-5-methoxybenzene (**3**), 1-[(7R)-hydroxy-8-propenyl]-3-[3′-methoxy-1′-(8′-propenyl)-phenoxy]-4,5-dimethoxybenzene (**4**), dihydrodieugenol (**5**), and dehydrodieugenol dimethyl ether (**6**) as shown in Fig. 2.

All isolated compounds were evaluated against promastigotes and amastigote forms of *L.* (L.) *infantum* to determine their EC_50_ values, followed by comparison of the values obtained for the standard drug (miltefosine). Furthermore, the cytotoxicity values (CC_50_) against NCTC – L929 cells as well as their in vitro selectivity index (SI) were determined. Promastigotes of *L.* (L.) *infantum* were incubated with compounds **1–6** for 48 h and the viability was determined by the MTT assay [10, 19, 20]. The obtained results indicated that compounds **4** and **6** were active, displaying EC_50_ values of 65.4 μM (95% confidence interval of 59.7 to 72.3 μM) and 88.8 μM (95% confidence interval of 76.2 to 103.7 μM), respectively. When tested against intracellular amastigotes, compounds **2**, **4** and **6** showed activity with EC_50_ values of 57.9 μM (95% confidence interval of 47.8 to 70.2 μM), 67.7 μM (95% confidence interval of 54.5 to 84.2 μM) and 13.7 μM (95% confidence interval of 11.7 to 16.0 μM), respectively. Compounds **1**, **3**, **5** and monomers (eugenol and methyl eugenol) showed no activity against either tested form at the maximum tested concentration (100 μM). Miltefosine was used as standard drug with EC_50_ values of 16.9 μM (95% C.I. = 11.6 to 18.6 μM) against promastigotes and 16.4 μM (15.5 to 17.5 μM) against intracellular amastigotes (Table 1). In the cytotoxicity assay against NCTC cells, compound **5** was toxic with a CC_50_ value of 58.1 μM, while no toxicity was observed in relation to the other compounds (CC_50_ >  200 μM). The standard drug miltefosine showed a CC_50_ value of 122 μM. Therefore, calculated SI values against amastigote forms were >  3.4, > 2.9 and >  14.6 for compounds **2**, **4** and **6** respectively.

**Table 1:** Anti- *Leishmania (L.) infantum* (promastigote and amastigote forms) and mammalian cytotoxicity (NCTC cells) activities of compounds **1–6** isolated from *Nectandra leucantha* and eugenol/methyleugenol

Compounds	EC_50_ (μM) 95% CI		CC_50_ (μM) 95% CI	SI Amastigote
	*Leishmania* (L.) *infantum*		NCTC	
	Amastigote	Promastigote		
**1**	NA	NA	> 200	–
**2**	57.9 (47.8–70.2)	NA	> 200	> 3.4
**3**	NA	NA	> 200	–
**4**	67.7 (54.5–84.2)	65.4 (59.7–72.3)	> 200	> 2.9
**5**	NA	NA	58.1	–
**6**	13.7 (11.7–16.0)	88.8 (76.2–103.7)	> 200	> 14.6
eugenol	NA	NA	> 200	–
methyleugenol	NA	NA	> 200	–
miltefosine	16.9 (11.6–18.6)	16.4 (15.5–17.5)	122. 0	7.2

EC_50_ 50% effective concentration, CC_50_ 50% cytotoxic concentration, SI selectivity index, NA Not Active 95%, CI 95% confidence interval

It is important to highlight that the efficacy and toxicity of compound **6** was similar to miltefosine. Recently, the DNDi (Drugs for Neglected Diseases initiative) defined criteria and proposed cutoff values to qualify the compounds investigated into hit compounds. They include: EC_50_ < 10 μM, SI tenfold more active vs. mammalian cell line, no structural alerts (metabolism, stability and reactivity), chemical tractability (acceptable synthetic pathway for compound and/or analogues (< 8 steps) etc. [21]. Compound **6** conforms with many of these criteria and could be eventually be addressed to the hit-to-lead optimization phase. Although the activity of compound **6** against promastigote forms of *L.* (L.) *amazonensis* has previously been reported in the literature [18], the present work described the activity of compound **6** against intracellular amastigotes of *L.* (L.) *infantum*, the clinically relevant form of *Leishmania*, which results in a fatal visceral disease if untreated [22].

Comparing the data shown in Table 1 with the chemical structures of compounds **1–6** and eugenol/methyleugenol, it was possible to suggest that coupling of monomers is a crucial step to obtain antileishmanial compounds since only dimeric derivatives (neolignans) displayed activity. By analyzing the activity data of closely related compounds **1–4**, it was revealed that compounds **2** and **4** were active, suggesting that the presence of a methoxyl group at C-4 constitutes an important chemical feature to enhance the antiparasitic potential. As observed, neolignans containing free hydroxyl group at C-4 (**1** and **3**) were inactive, suggesting that polarity directly affects the antileishmanial potential of related compounds. Similarly, the presence of an additional hydroxyl group in the allyl side chain, as observed in compounds **2** and **4**, causes a reduction in the potential. Comparing the activity of neolignans **5** and **6**, it was observed that the presence of additional methoxyl group also enhances the antileishmanial activity as displayed by compounds **1–4**, suggesting that the presence of this substituent is crucial to antileishmanial activity of these related neolignans. Finally, as previously reported [13], in silico analysis of compounds **1–6** indicated that compound **6** was predicted as a non pan-assay interference compound (PAINS) and displayed some favorable in silico ADMET properties - non-mutagenic, non-carcinogenic, non-genotoxic, weak hERG blockers, with an acceptable volume of distribution (1.66–3.32 L/kg), and low rodent oral toxicity (LD_50_ = 810–2200 mg/kg).
